# Is regional atrial strain a useful surrogate of regional atrial fibrosis in atrial cardiomyopathy?

**DOI:** 10.1093/ehjimp/qyaf068

**Published:** 2025-05-28

**Authors:** Angela W C Lee, Charles Sillett, José Alonso Solis-Lemus, Cassia Kessler Iglesias, Luuk H G A Hopman, Alina Hua, Peter Wheen, Abdul Qayyum, Marina Strocchi, Caroline Roney, Thomas Booth, Tevfik F Ismail, Henry Chubb, Daniel B Ennis, Andrew Jabbour, Diane Fatkin, Marco J W Götte, Steven A Niederer

**Affiliations:** School of Biomedical Engineering and Imaging Sciences, King’s College London, St Thomas’ Hospital, London, UK; National Heart and Lung Institute, Imperial Centre for Translational and Experimental Medicine, Imperial College London, 72 Du Cane Rd, London W12 0NN, UK; School of Biomedical Engineering and Imaging Sciences, King’s College London, St Thomas’ Hospital, London, UK; National Heart and Lung Institute, Imperial Centre for Translational and Experimental Medicine, Imperial College London, 72 Du Cane Rd, London W12 0NN, UK; School of Biomedical Engineering and Imaging Sciences, King’s College London, St Thomas’ Hospital, London, UK; National Heart and Lung Institute, Imperial Centre for Translational and Experimental Medicine, Imperial College London, 72 Du Cane Rd, London W12 0NN, UK; Victor Chang Cardiac Research Institute, Darlinghurst, New South Wales, Australia; Cardiology Department, St Vincent’s Hospital Sydney, Darlinghurst, New South Wales, Australia; School of Clinical Medicine, Faculty of Medicine and Health, UNSW Sydney, Kensington, New South Wales, Australia; Department of Cardiology, Amsterdam University Medical Center, Amsterdam, The Netherlands; School of Biomedical Engineering and Imaging Sciences, King’s College London, St Thomas’ Hospital, London, UK; Cardiology Department, Guy’s and St Thomas’ NHS Foundation Trust, London, UK; School of Biomedical Engineering and Imaging Sciences, King’s College London, St Thomas’ Hospital, London, UK; Cardiology Department, Guy’s and St Thomas’ NHS Foundation Trust, London, UK; National Heart and Lung Institute, Imperial Centre for Translational and Experimental Medicine, Imperial College London, 72 Du Cane Rd, London W12 0NN, UK; National Heart and Lung Institute, Imperial Centre for Translational and Experimental Medicine, Imperial College London, 72 Du Cane Rd, London W12 0NN, UK; School of Biomedical Engineering and Imaging Sciences, King’s College London, St Thomas’ Hospital, London, UK; School of Engineering and Materials Science, Queen Mary University of London, London, UK; School of Biomedical Engineering and Imaging Sciences, King’s College London, St Thomas’ Hospital, London, UK; Department of Neuroradiology, Kings College Hospital, Ruskin Wing, London, UK; School of Biomedical Engineering and Imaging Sciences, King’s College London, St Thomas’ Hospital, London, UK; Cardiology Department, Guy’s and St Thomas’ NHS Foundation Trust, London, UK; Division of Pediatric Cardiology, Department of Pediatrics, Stanford University, Stanford, California, USA; Division of Pediatric Radiology and Cardiovascular Imaging, Stanford University, Stanford, California, USA; Victor Chang Cardiac Research Institute, Darlinghurst, New South Wales, Australia; Cardiology Department, St Vincent’s Hospital Sydney, Darlinghurst, New South Wales, Australia; School of Clinical Medicine, Faculty of Medicine and Health, UNSW Sydney, Kensington, New South Wales, Australia; Victor Chang Cardiac Research Institute, Darlinghurst, New South Wales, Australia; Cardiology Department, St Vincent’s Hospital Sydney, Darlinghurst, New South Wales, Australia; School of Clinical Medicine, Faculty of Medicine and Health, UNSW Sydney, Kensington, New South Wales, Australia; Department of Cardiology, Amsterdam University Medical Center, Amsterdam, The Netherlands; School of Biomedical Engineering and Imaging Sciences, King’s College London, St Thomas’ Hospital, London, UK; National Heart and Lung Institute, Imperial Centre for Translational and Experimental Medicine, Imperial College London, 72 Du Cane Rd, London W12 0NN, UK; Digital Twin Turing Research and Innovation Cluster, Alan Turing Institute, London, UK

**Keywords:** atrial fibrillation, cardiac magnetic resonance, 3D atrial strain, atrial fibrosis

## Abstract

**Aims:**

To determine whether atrial biomechanics measured using 3D regional strain, left atrial volume (LAV), and left atrial emptying fraction (LAEF) are associated with atrial fibrosis in patients with suspected atrial cardiomyopathy.

**Methods and results:**

Cardiovascular magnetic resonance (CMR) was performed in atrial fibrillation (AF) patients (*n* = 47). Healthy volunteer (*n* = 41) and familial dilated cardiomyopathy (DCM) (*n* = 31) cohorts were acquired for normalization and validation, respectively. Fibrosis was quantified using late gadolinium enhancement (LGE)-CMR, and 3D regional strain was quantified using feature tracking. Machine learning classifiers were used to classify regional severe fibrosis (>30% LGE enhancement) using regional strain and global measures of atrial anatomy and function. 3D regional strain measures (peak reservoir strain or first/second strain principal component) alone were not associated with regional fibrosis (accuracies ≤ 56.0%) in the AF cohort. Severe fibrosis was found primarily in the lateral (85.1% of AF patients) and posterior (66.0%) regions. In AF patients, the classifier incorporating LAV, LAEF, and regional location was associated with severe regional fibrosis (AUC = 0.86 ± 0.06, accuracy = 79.4 ± 6.2%), while in familial DCM patients, the accuracy was lower (62.8%).

**Conclusion:**

There is a distinctive pattern of fibrosis that develops with progression of atrial cardiomyopathy, irrespective of cause. Global measures reflecting overall atrial anatomy (LAV) and function (LAEF), rather than localized regional 3D strain, were associated with severe regional fibrosis. These data suggest that regional atrial 3D strain alone is not a reliable surrogate for severe regional fibrosis.

## Introduction

Atrial fibrillation (AF) is a complex and progressive disease with left atrial (LA) enlargement, contractile dysfunction and fibrosis contributing to the underlying substrate that initiates and maintains AF.^1^ However, the relationship between the LA anatomy, function, and structure remains largely unclear. This is an important unresolved issue and may provide clues to AF treatment and prevention.

Cardiovascular magnetic resonance (CMR) imaging is used extensively to characterize atrial anatomy, strain and fibrosis using contrast enhanced magnetic resonance angiography (CE-MRA), cine and late gadolinium enhancement (LGE-CMR)^2,3^ sequences, respectively. Global fibrosis burden from LGE enhancement has been found to predict AF recurrence after ablation.^2,3^ Subsequent studies indicated that the regional location of fibrosis may be a better predictor^4,5^ and may be useful to guide AF ablation.^6,7^ Increased LA volume (LAV) is an important indicator of prognosis, associated with higher fibrosis burden and increased likelihood of AF recurrence after ablation.^8–10^ LA function can also be measured with LA strain, facilitating a better characterization of LA compliance and contractile function. Studies have shown that global strain measures correlates with the total fibrosis burden and AF recurrence,^11,12^ while 2D regional strain in two-chamber and four-chamber CMR images was found to be correlated with regional fibrosis.^13^ A strong correlation between localized strain and fibrosis would support the potential use of strain assessment as a non-invasive marker of fibrosis. Changes in 3D regional strain could be useful in identifying fibrosis on a per-patient basis and thus could potentially have clinical utility in risk stratification for AF patients. However, this would require a substantial, clinically meaningful change.

We sought to determine whether atrial biomechanics measured using 3D measures of regional strain, LAV, and left atrial emptying fraction (LAEF) were associated with fibrosis burden in AF patients and in patients with suspected atrial cardiomyopathy due to familial dilated cardiomyopathy (DCM).

## Methods

### Study cohorts

CMR images were acquired from patient populations and healthy subjects from three centres during sinus rhythm (*Table 1*).

**Table 1:** Characteristics of the three distinct populations studied

	Healthy (*n* = 41)	AF (*n* = 47)	DCM (*n* = 31)
Age	32.2 ± 7.2	60.0 ± 8.4	47.4 ± 16.2
Gender (male)	(32) 78%	(31) 66%	(13) 42%
LVEF (%)	60.0 ± 4.1	59.0 ± 8.2	56.2 ± 5.6
LAEF (%)	60.6 ± 6.3	51.1 ± 14.3	58.2 ± 10.3
Paroxysmal/persistent AF	N/A	(33/14) 70%/30%	(1/0) 3%/0%

A healthy volunteer cohort, an atrial fibrillation (AF) patient cohort, and a familial dilated cardiomyopathy (DCM) cohort.

LAEF, left atrial emptying fraction; LVEF, left ventricular ejection fraction.

An AF cohort (*n* = 47, 60 ± 8.4 years, 66% male) was recruited at the University Medical Center Amsterdam (Amsterdam, The Netherlands). Patients had paroxysmal (*n* = 33) or persistent AF (*n* = 14), were scheduled to undergo their first pulmonary vein isolation, and were in sinus rhythm during the CMR scan (see Supplement for clinical characteristics). A healthy cohort (*n* = 41, 32 ± 7.7 years, 78% male) was recruited at Guy’s and St Thomas’ NHS Foundation Trust (London, UK) and underwent CMR imaging without contrast to provide a control dataset. A third population of probands and relatives with familial DCM (*n* = 31, 47.4 ± 16.2 years, 42% male) was recruited at the Victor Chang Cardiac Research Institute (Sydney, Australia) for CMR imaging as a validation dataset.

### CMR methods

In the AF cohort, cine axial CMR image stacks covering the LA (in-plane resolution: 0.7–0.8 mm^2^, 8 mm slice thickness, 30 timeframes) were acquired on a Siemens 1.5T Avanto scanner (Siemens Healthineers, Erlangen, Germany). CE-MRA and LGE-CMR images were simultaneously acquired (in-plane resolution: 0.625 mm^2^, 1.25 mm slice thickness). In the healthy volunteer cohort, cine short axis CMR images were acquired (in-plane resolution: 1.6–2 mm^2^, slice thickness 4–8 mm, and 20–50 timeframes) using Siemens 1.5T MAGNETOM Aera or 3T VIDA scanners. In the familial DCM cohort, cine short axis CMR images (in-plane resolution: 1.2–1.5 mm^2^, 4–8 mm slice thickness, 25 timeframes) were acquired on a Siemens 3T Prisma scanner. In a subset of this cohort (*n* = 26, 49.2 ± 15.5 years, 38% male), LGE-CMR and CE-MRA images were simultaneously acquired. Of the 31 patients recruited, six patients were excluded due to lack of LGE-CMR or MRA images (*n* = 3; contrast allergies, technical errors, or claustrophobia); or artefacts arising from implanted cardioverter-defibrillators (*n* = 3).

### Data analysis

**Figure 1:**
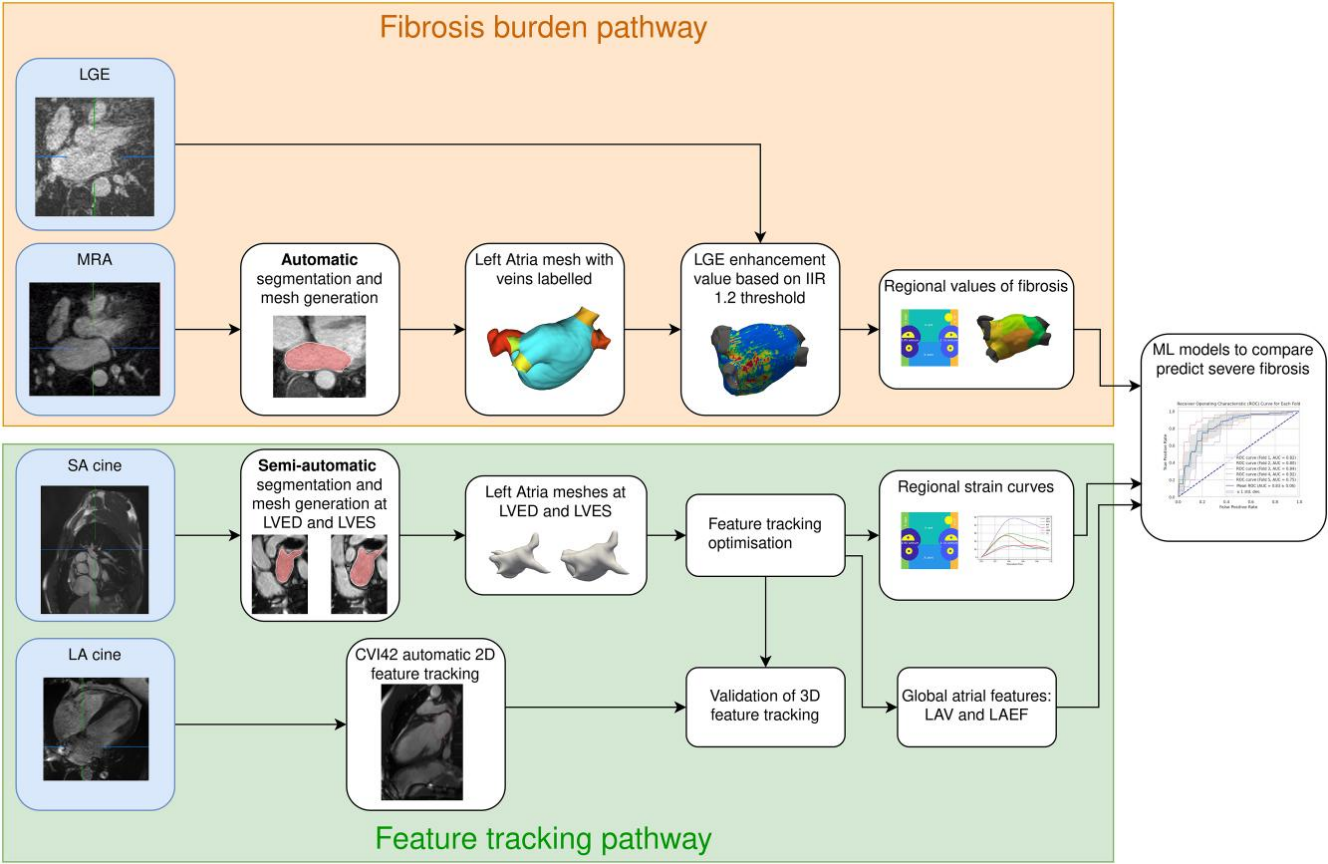
Modelling workflow. The overall workflow for determining the regional fibrosis and regional strain values for the left atria (LA) from cardiac magnetic resonance (CMR) imaging. In the fibrosis pathway, CE-MRA images are automatically segmented and used to generate a mesh. The fibrosis burden in the LA is defined with the LGE-CMR images. The feature tracking pathway uses the 3D cine MR images to track the LA motion throughout the cardiac cycle. Severe regional fibrosis was classified with machine learning (ML) models using regional information (region location and strain) and 3D global features (LA volume and LA emptying fraction).

Anatomical models of the LA were generated to track 3D cardiac motion and simultaneously identify regions of fibrosis in the LA. The workflow for comparing regional strain against fibrosis is shown in *Figure 1*. For fibrosis quantification, the LA blood pool was semi-automatically segmented and labelled using a convolutional neural network from the CE-MRA images using CemrgApp (cemrgapp.com).^14^ A surface mesh of the LA, with the pulmonary veins (PV) and left atrial appendage (LAA) labelled, was generated.^14^ An image intensity ration (IIR) threshold of 1.2 was used to define fibrosis burden from the LGE-CMR images.^15,16^ The % area of enhancement in the LGE-CMR image was used to differentiate between stages of fibrosis burden (1: <10% area of LGE enhancement, 2: 10–20%, 3: 20–30%, 4: >30%).^3^

**Figure 2:**
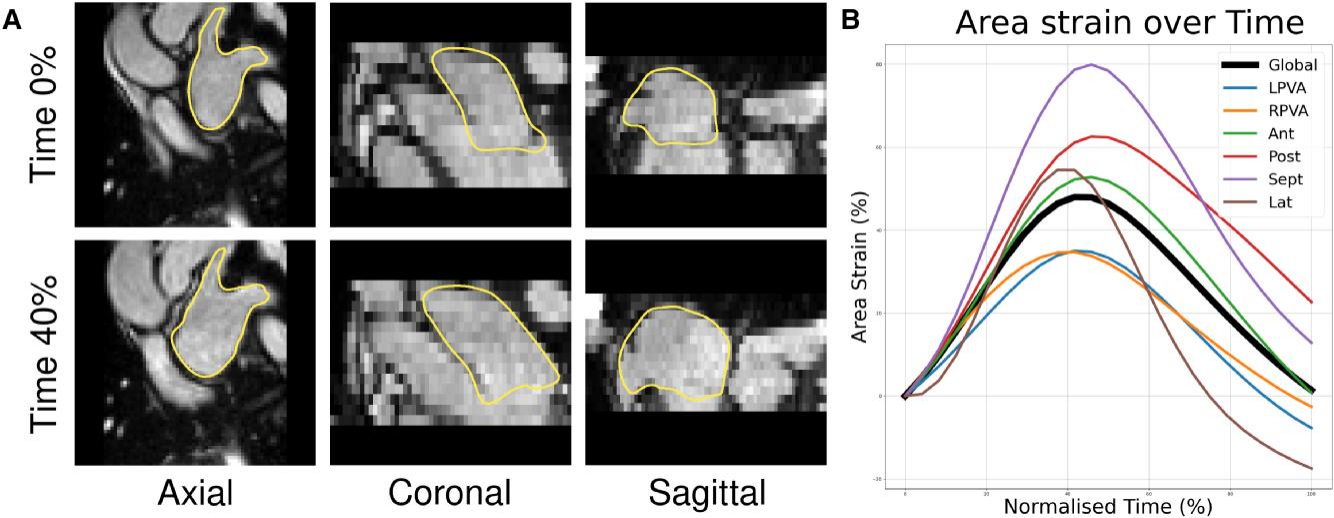
(*A*) The feature tracking algorithm was used to track the deformation of the atria throughout the cardiac cycle. The overlay outline shows the deformation of the mesh from time 0% to time 40% of the cardiac cycle. (*B*) A representative strain curve showing the global and regional area strains throughout the cardiac cycle. Ant, anterior wall; Lat, left lateral wall; LPVA/RPVA, left/right pulmonary venous antrum; Post, posterior wall; Sept, septum.

For the feature tracking pathway (*Figure 1*), LA blood pool in the cine MR images were segmented manually using the region growing segmentation tool in Medical Imaging and Interaction Framework (MITK, www.mitk.org) at left ventricular end-diastolic (LVED) and end-systolic (LVES) timepoints. Strain curves were calculated using the temporal sparse free form deformation (TSFFD) method to track the cardiac motion (*Figure 2*), which has previously been used to track LA motion in CT and RA motion in CMR.^17,18^ The LAA and PV were excluded to calculate the 3D LAV and LAEF.

**Figure 3:**
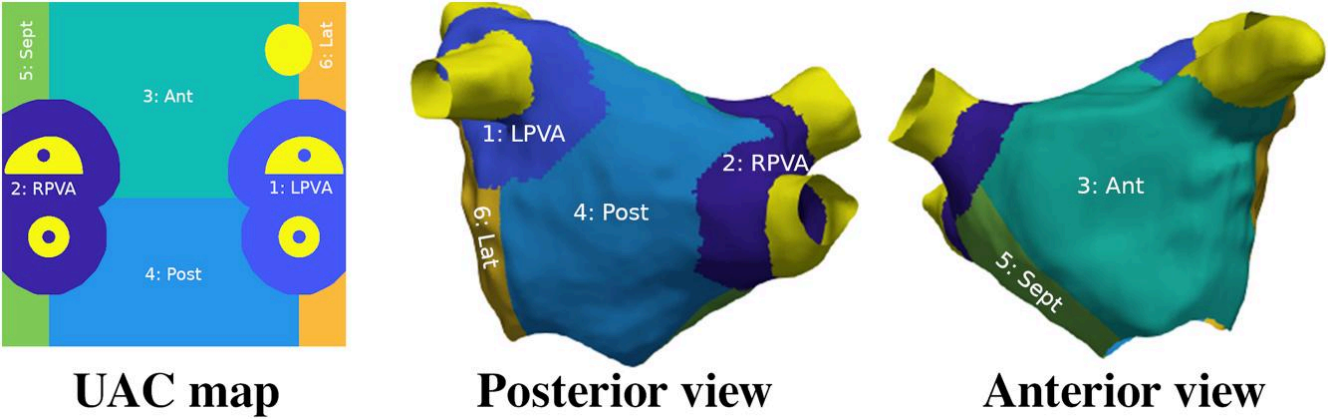
UACs were used to map regions in the 3D LA meshes. Ant, anterior wall; AP view, anterior posterior view; Lat, left lateral wall; LPVA/RPVA, left/right pulmonary venous antrum; PA view, posterior anterior view; Post, posterior wall; Sept, septum.

Universal atrial co-ordinates (UAC) were mapped onto the LA meshes.^17^ The UACs were used to subdivide the LA: left/right PV antrums, posterior, septal, anterior, and left lateral walls (*Figure 3*).^19^ The PV antra were defined as 10 mm from the PV–LA body junction.^19^ Endocardial and epicardial fibre fields derived from a human atlas were mapped via UACs to test the effects of fibre directions on strain (see Supplement for details).^20^

The function of the heart was measured using the regional and global area strains, initialized at the LVED timepoint. The reservoir strain (RS) was taken as the amplitude of the strain curves. To impartially identify uncorrelated features [principal component (PC)] of the strain curves, we applied a PC analysis (PCA) to decompose the strain curves into a lower dimensional space (see Supplement for details).^21^

Atrial strain is very heterogenous, relative to the ventricle, and may need to be normalized. *Z*-scores (*Z*_AF_) were calculated with the RS (*x*_AF_) of the AF cohort using the mean (*μ*_Healthy_) and SD (*σ*_Healthy_) of the healthy cohort RS:


$$Z_{\mathrm{AF}}=(x_{\mathrm{AF}}-\mu_{\mathrm{Healthy}})/\sigma_{\mathrm{Healthy}}.$$

Machine learning (ML) models were used to investigate if features or a combination of features were able to classify regions with stage 1–3 fibrosis or stage 4 (severe fibrosis)^3^ in the AF cohort. Given the modest sample size of the AF patient cohort (*n* = 47), ML was used as a hypothesis testing method rather than as a proposed clinical classification tool. To test if a relationship between atrial strains and fibrosis depended on regional and global measures of atrial disease progression, we used Random forest (RF) classifiers. Regional (LA region, regional area RS, regional area strain rate, PCs of the strain curves) and global (area RS, LAEF and LAV calculated from the 3D mesh) features were considered. Logistic Regression and AdaBoost techniques were also tested (see Supplement).

To assess the accuracy of the model, we split the AF patient data (*n* = 47) into training (80%) and validation (20%) datasets. Five-fold cross-validation with an RF model was used to classify regions of severe fibrosis. Regional features assessed were area RS, area strain rate, the first and second PCs of the strain curves, and the location of the region. Global features (area RS, 3D LAEF, and LAV) were also assessed. Finally, to assess the robustness of our methods, we tested the final model in an independent disease cohort with familial DCM, in which changes in atrial size and function were anticipated.

#### Statistical methods

Comparisons of the fibrosis burden or the area strain across the atrial regions were performed using one-way ANOVA, followed by *post hoc* Tukey tests. Mixed effects models were used to assess the impact on area strain of the regional location combined with either severe fibrosis classification or cohort classification (healthy vs. AF). Significant differences (*P* < 0.05) are presented in the results section with full analysis detailed in the Supplement.

## Results

### Quantification of fibrosis

**Figure 4:**
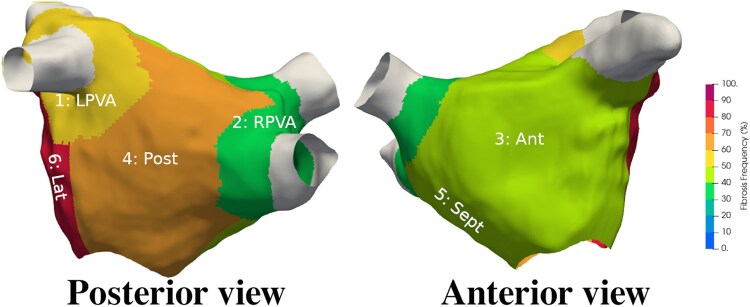
The regional frequency of severe fibrosis in the atrial fibrillation (AF) patient cohort. The highest probability of severe fibrosis in the whole AF cohort was seen in the lateral and posterior regions. LPVA/RPVA, left/right pulmonary venous antrum; Ant, anterior wall; Post, posterior wall; Sept, septum; Lat, left lateral wall.

Significant regional differences in atrial fibrosis burden were identified in AF patients (one-way ANOVA, *P* < 0.001), where we have used a threshold of 30% area enhancement to differentiate between regions with severe fibrosis (61.7% of regions) or not (38.3%).^3^ In a significant majority, there was severe fibrosis in the lateral (85% of patients) and posterior (66%) regions (*Figure 4*). The fibrosis burden was significantly higher (*P* < 0.05) in the lateral (44.4 ± 17.2%) compared with LPVA (26.0 ± 18.5%, *P* < 0.001), anterior (31.4 ± 14.9%, *P* = 0.011), and septal (32.7 ± 20.1%, *P* = 0.031) regions. There were also significant differences between the LPVA and posterior regions (40.9% ± 21.8%, *P* = 0.002).

### Is regional strain associated with fibrosis burden?

The association between regional area strain and fibrosis was tested in the AF cohort data using mixed effects models. Fibrosis classification did not have a significant effect on area strain accounting for the regional locations (*P*-values 0.296–0.872) or without (*P* = 0.774). Similarly, fibrosis classification had no significant effect PCs of the area strains curves (PC1 *P* = 0.860, PC2 *P* = 0.218) (see Supplement for details).

### Are relative regional strain changes associated with fibrosis burden?

Regional strains were found to be 48.0 ± 33.2% and 19.5 ± 15.7% in the healthy and AF cohorts, respectively. There was significant heterogeneity in the area strains across the regions within each cohort (one-way ANOVA, *P* < 0.001) with significantly higher area strain in the septal regions (*P* < 0.001). The largest and smallest relative strains were in the septal and the PVA (RPVA for healthy and LPVA for AF), respectively.

The overall regional area strain was significantly lower in the AF patient cohort (*P* < 0.001). To account for these physiological strain variations, we calculated the *Z*-scores in AF patients relative to the healthy volunteer strains. We found that the fibrosis burden had no significant effect on the relative area strain (*P* > 0.6).

### Is there a combination of factors that are associated with severe fibrosis?

**Figure 5:**
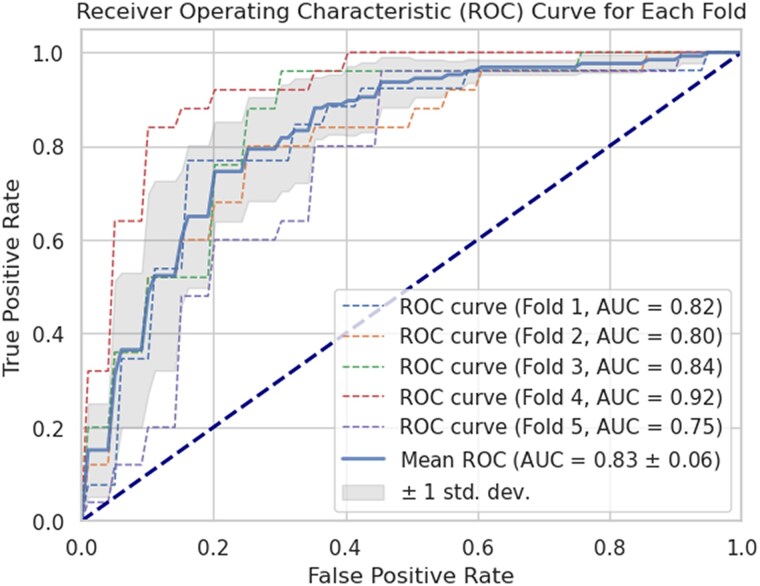
The ROC curves for the five-fold cross-validation of the optimal classifier [left atrial (LA) volume, LA emptying fraction, and regional location] to classify severe fibrosis in the atrial fibrillation patient cohort.

To test if a relationship between atrial strains and fibrosis depended on regional and global measures of atrial disease progression, we used RF models to assess the ability of different features in classifying severe regional fibrosis in the AF cohort. Regional (LA region, area and fibre RS, PCs of the strain curves) and global (LAEF and LAV calculated from the 3D mesh) features were considered. We found that the features that were able to classify regional severe fibrosis better than random chance (*P* < 0.05) were the region (*P* = 0.044), PC2 of the regional area strain curve (*P* = 0.036) and the global area RS (*P* = 0.003), LAEF (*P* = 0.001), and LAV (*P* = 0.002), with the global features having the highest accuracy (global area RS: 75 ± 8%, LAEF: 76 ± 6%, and LAV: 76 ± 7%) compared with the regional features having accuracies ≤ 56.0% (*Table 2*). The combination of features was found to incrementally improve the accuracy of the classifier. The first order interactions of the features were examined, and we observed that the combination of regional location, LAV, and LAEF had accuracies > 70% (*Table 3*). The combination of all three factors (LAEF + LAV + region) had an accuracy of 79.4 ± 6.2% in classifying severe fibrosis in the AF cohort, with a ROC AUC = 0.83 ± 0.06 (*Figure 5*), indicating that there is a common pattern of severe fibrosis that develops with LA remodelling.

**Table 2:** Machine learning models were used to classify severe fibrosis in atrial fibrillation patients

	Five-fold cross-validation accuracy (%)	Classifier *P*-value	ROC AUC	ROC AUC *P*-value
Regional				
Region	**56.0** ± **4.0**	**0**.**044**	**0**.**638**	**0**.**027**
Area RS	53.2 ± 5.4	0.307	0.506	0.500
Area PC1	49.0 ± 7.0	0.860	0.361	0.974
Area PC2	**56.0** ± **3.9**	**0**.**036**	**0**.**666**	**0**.**018**
Area strain rate	45.3 ± 6.7%	0.792	0.441	0.763
Global				
3D LAEF	**75.9** ± **5.8**	**0**.**001**	**0**.**797**	**0**.**001**
3D LAV	**76.6** ± **7.2**	**0**.**002**	**0**.**799**	**0**.**001**
Area RS	**74.8** ± **7.5**	**0**.**003**	**0**.**811**	**0**.**001**

Regional and global features were tested and models that do better than pure chance (*P* < 0.05) are bolded.

RS, reservoir strain; PC1/2, first/second principal components of the strain curves; LAEF, left atrial emptying fraction; LAV, left atrial volume.

**Table 3:** Five-fold cross-validation accuracy of the combination of regional and global features in classifying severe fibrosis in the atrial fibrillation patient cohort

	3D LAEF	3D LAV	Area PC2	Global area RS
Region	71.0 ± 3.0%	72.0 ± 7.0%		69.5 ± 7.4%
LAEF		77.3 ± 6.5%	66.0 ± 5.0%	74.5 ± 6.2%
LAV			66.0 ± 2.0%	73.8 ± 7.4%
Area PC2				63.4 ± 6.3%

The models that do no better than pure chance (*P* > 0.05) are excluded from the table.

PC2, second principal components of the strain curves; LAEF, left atrial emptying fraction; LAV, left atrial volume.

The workflow was applied to a familial DCM cohort to test the classifier in an independent dataset and assess if they generalized beyond patients indicated for AF ablation. Histological studies have shown high prevalence of LA fibrosis in patients with familial and sporadic DCM.^22^ Moreover, patients with genetically mediated ventricular cardiomyopathy frequently have primary or secondary changes in atrial size and/or function.^23–25^ In the familial DCM cohort, 11 had DCM and 14 were genotype-positive without overt DCM. There were nine patients with atrial dilation (left atrial volume index [LAVI] > 53 mL/m^2^),^(26)^ of whom seven had DCM, and one patient (with DCM and atrial dilation) had paroxysmal AF. Severe regional fibrosis was seen in 10 patients, four of whom had DCM. We tested the optimal classifier (LAEF + LAV + region) and found that we were able to classify severe fibrosis in each region with 62.8% accuracy for the familial DCM cohort.

## Discussion

We have investigated the link between LA biomechanics and regional fibrosis. LGE-CMR studies have found that the regional distribution of fibrosis to be predictive of AF recurrence after ablation,^4,5^ and has been suggested as a biomarker for AF ablation targets.^6,7^ Demonstration of a strong correlation between localized fibrosis and strain measures could potentially provide an alternative measure of risk stratification for AF ablation. In this study, we developed and applied a novel image analysis workflow to measure 3D atrial area strains and provide reference strain measures in human subjects. We have shown that multiple measures of regional strain are not associated with regions of LA fibrosis. This agrees with Peters *et al*.,^13^ who found that 2D regional strain did not classify fibrosis better than pure chance (ROC AUC = 0.66, *P* = 0.11). We found that regional fibrosis was best classified based on the region and global measures of LA size and function in AF patients and we tested this classifier in a familial DCM cohort.

Increased collagen deposition is expected to increase tissue stiffness that in turn should impact regional cardiac biomechanics.^27^ While an association has been shown for LV fibrosis and regional strain,^28^ we found no such association in the LA. In healthy control subjects, the heterogeneity of longitudinal and circumferential LA strains was found to be higher [standard deviation (SD): 9.2% and 13.4%, respectively] than LV strains (SD: 2.9% and 3.1%, respectively).^29^ This higher variability in strains may make it harder to identify deviations from the norm that can potentially be used to classify regional fibrosis. The lack of a link between any of the regional measures of atrial strain and fibrosis in our study is surprising, but three potential explanations could be considered. First, atrial strain may be influenced predominantly by the complex atrial anatomy, heterogenous wall thickness,^30^ in addition to loading from the ventricle,^31^ with Cameli *et al*.^32^ finding that atrial strain was a good correlate of the left ventricular end diastolic pressure (LVEDP). Thus changes in regional fibrosis burden may have a limited or secondary effect on regional strain. Secondly, we did not differentiate between dense and patchy fibrosis that may have distinct impacts on regional biomechanics.^33^ Thirdly, in a simplified spherical model of the atria, strain is proportional to pressure and the radius, and inversely proportional to wall thickness and stiffness.^34^ During AF, pressure, radius (as a measure of volume), and stiffness (due to fibrosis) all potentially change. This multifactorial relationship may obfuscate a simple link between fibrosis and its expected effect on strain. To quantify the impact of fibrosis on regional atrial biomechanics may require additional measurements (pressure and wall thickness)^34^ and biomechanical models to estimate regional stiffness, while accounting for complex atrial anatomy and ventricular loading.

We found that there was a common pattern of severe regional fibrosis, consistent with previous studies.^16,19,35^ While specific regions used in each study vary, when accounting for these different regions (see Supplement), we see a consistent pattern across studies. In our AF cohort, the lateral and posterior regions had the highest likelihood of severe fibrosis, while LPVA had the least. This is consistent with previous findings of high posterior fibrosis,^35^ but contrasts with the finding of high fibrosis around the LA PV.^17,26^ However, this may be due to differences in anatomical regions definitions. Currently, there is no consensus on the definition of regions in the LA, and while we have used the Higuchi mapping,^19^ other models such the Benito mapping^16^ are equally valid. Using the Benito mapping, we found that the AF cohort had a significant level of fibrosis in the posterior wall subdivisions adjacent to the left inferior pulmonary vein (LIPV), consistent with Benito *et al.* findings (see Supplement).^16^ In our AF cohort, there were additional regions with increased fibrosis burden, namely the floor, left lateral wall, and anterior regions close to the mitral valve. Atrial anatomical regions definitions can impact findings, and a community standard would facilitate study replication. However, the present study is the first to show that this fibrosis pattern appears to develop as the atria remodels, that the pattern, to an extent, is common across AF and non-AF cases, and can be classified based on the region, LAEF, and LAV.

We found that global measures of atrial anatomy (LAV) and function (LAEF) were significantly associated with regional severe LA fibrosis burden in our AF and familial DCM cohorts, consistent with reported correlations between global fibrosis burden and LA structure and function.^34,36,37^ These results are consistent with common drivers of fibrosis in the atria that are not unique to AF patients. The ability of our classifier to identify regions of severe fibrosis, independent of disease pathology, indicates that the link between the global function and size of the atria and fibrosis burden is an intrinsic property of the atria rather than a process driven by AF.^38^ Not all studies found a link between LAV and LAEF with regional or global fibrosis burden.^39^ This may be due to different thresholds to define severe fibrosis, the use of correlations, as opposed to ML, to identify relationships, or estimation of LAEF from 2D long axis cine MRI. However, in general, there is a consistent finding that atrial fibrosis increases in line with atrial dilation and compromised function.^16,19,38–40^

## Study limitations

Our study had several limitations. First, the relatively small number of AF patients may limit our ability to measure subtle relationships between regional fibrosis and strain, and despite finding no correlation in this study, a weaker link may still be present. While increasing the number of participants could potentially identify a statistically significant difference in the strains in severely fibrotic regions, the large variance in regional strains means that the difference may not be practically significant or useful clinically as a surrogate in identifying localized fibrosis on a per-patient basis. Secondly, the healthy cohort we used to normalize atrial strains in the AF cohort was not demographically matched. As atrial anatomy and, likely strains, change with age and body surface area, this may have impacted the results. In addition, the CMR images were acquired from different centres, protocols, and scanner types that could have further confounded differences between the healthy and AF cohorts. Thirdly, the cine MR images for the AF patient cohort were acquired during sinus rhythm. While this improves image quality, we recognize that this may not fully capture the underlying pathophysiology in patients with more irregular or advanced AF, potentially reducing the generalizability of our methods. The observed strain differences may also reflect broader disease-related factors—such as reduced atrial contractility or elevated left ventricular filling pressures—rather than fibrosis alone. Although we calculated *Z*-scores relative to healthy volunteers to account for physiological variation, this approach did not yield clearly discriminative patterns. Additionally, the use of k-fold cross-validation within a single AF dataset may overestimate classifier performance and limit generalizability. Finally, the familial DCM cohort was also recruited from a separate centre with a different scanner and protocol, while this allows us to demonstrate the generalization of our methods, where classifier was still able to identify regions with severe fibrosis, this may have impacted on the accuracy of our classifier. The reduction in accuracy in comparison to the AF cohort may also reflect the heterogeneity of the LA pathology in the familial DCM cohort and that other factors could also be associated with severe fibrosis in this cohort.

## Clinical perspectives

In our study, we found that regional LA strain was not well associated with regional LA fibrosis. The pattern of LA regional fibrosis can best be classified from the extent of atrial cardiomyopathy as assessed by LAV and LAEF. This relationship was similar in patients with and without a history of AF.

## Supplementary Material

qyaf068_Supplementary_Data

## Data Availability

The datasets presented in this article are not readily available to be shared publicly due to the sensitive nature of patient data. Reasonable requests to access the datasets should be directed to the corresponding author, A.W.C.L.

## Lead author biography

Dr Angela W.C. Lee received her PhD in Biomedical Engineering from the University of Auckland, New Zealand. Her main research focus is on using patient-specific models of the heart to investigate dyssynchrony in the heart. Previously, her work focused on modelling the chronic changes in the heart’s electrophysiology, mechanics, and geometry due to cardiac resynchronization therapy and the effect that these changes will have on the optimal lead location. The current focus of her work is on modelling fibrosis in the left atria and its correlation with atrial biomechanics and electrophysiological changes in the heart.
