# Supplementary material for: Is regional atrial strain a useful surrogate of regional atrial fibrosis in atrial cardiomyopathy?
Source: Eur Heart J Imaging Methods Pract. 2025 May 28;3(2):qyaf068. doi: 10.1093/ehjimp/qyaf068 (PMC12242763; doi:10.1093/ehjimp/qyaf068)
Supplement: qyaf068_Supplementary_Data [file qyaf068_supplementary_data.docx]

**Online Supplement**

**1. Clinical characteristics of the atrial fibrillation cohort**

A patient cohort of 47 atrial fibrillation (AF) patients were recruited between May 2019 and February 2020 at the University Medical Center Utrecht (Utrecht, Netherlands). All patients had paroxysmal or persistent AF as defined by the HRS/EHRA guidelines^1^ and were scheduled to undergo their first pulmonary vein isolation. The characteristics of the atrial fibrillation cohort are presented in the table below. LVEF, LAEF and LA volume index are calculated from the 2 chamber and 4 chamber CMR images.

*Table 1: Baseline clinical characteristics of the atrial fibrillation patient cohort.*

| Characteristic | Value |
| --- | --- |
| Age (years) | 60±8.45 |
| Men | 31(66%) |
| Body mass index (kg/m^2^) | 25.8±3.7 |
| Hypertension | 17 (36.2%) |
| Diabetes mellitus | 2 (4.3%) |
| History of stroke/TIA | 1 (2.1%) |
| CHAD2DS2-VASC score | 1.23±1.27 |
| Paroxysmal AF | 33 (70.2%) |
| LVEF (%) | 59.0±8.2 |
| LA volume index (mL/m^2^) | 50.1±16.3 |
| LAEF (%) | 51.1±14.4 |

*AF, atrial fibrillation; LAEF, left atrial emptying fraction; LVEF,* *left ventricle ejection fraction; LA, left atrium; TIA, transient ischaemic attack.*

**2. LA global features of healthy volunteers and atrial fibrillation patient cohorts**

Global features of the LA have been shown to change due to AF remodelling with an increased LAV and reduced LAEF. In this section, we present the LAV and LEAF for the AF patient and healthy volunteer cohorts. Pathological changes in the LA have been linked with an increasing LA size, with den Uijl et al showing that an increased LA size is correlated with AF recurrence and increased fibrosis burden.^2^ Comparison of the AF patient cohort with our healthy volunteer cohort has borne out this difference in with the AF patients having a significantly (p-value<0.001) larger LAV (3D: 111±36mL vs 78.4±26.9mL) and a reduced LAEF (22±9% vs 36±9%), as calculated from the 3D CMR image segmentations. This was found to be comparable to literature values of the healthy state of the LA (LAV: 67±20% and LAEF: 59±8%) and as AF progresses (LAV: 98±27mL to 141±39 mL, LAEF: 51±17% to 22%±11%).^3,4^

**3: Validation and verification of the TSFFD method for cine MR images**

Strain was measured with the widely used TSFFD cardiac CMR feature tracking algorithm (Figure 1). The TSFFD method was verified for the cine MR images used in the workflow, using the methods described by Sillett et al, 2024, where it was applied to retrospective gated CT images on the left atria^5^ Synthetic MR images were generated with known displacements applied to the ED MR image. We then used the TSFFD algorithm to register the ED MR image to the synthetic image and generate new displacements fields. The LA mesh could then be deformed with both displacement fields and the ASD between the 2 meshes can then be used to assess the accuracy and robustness of this method applied to cine MR images. The TSFFD verification found that for all 3 cohorts (healthy, AF and DCM), the mean ASD between the meshes at all time points for all patients in the cohorts were <2.5mm (healthy: 2.39mm±0.95mm; AF: 1.97mm±0.89mm; DCM: 2.18mm±0.89mm), consistent with image resolution.

We validated the TSFFD method by comparing the 3D motion tracking results against proprietary 2D motion tracking algorithms. Long axis (LAX) MR images slices in the 2 chamber (2ch) and 4 chamber (4ch) views were acquired alongside the cine images for the healthy and DCM familial cohorts. Circle CVI^42^ was used to automatically segment and track the cardiac motion of the LA in the 2D cine MR images in the 2ch and 4ch slices. Global measures of cardiac function of the LVEF, LAEF, and LAV were automatically calculated with this software from the long axis images. The contours characterizing the motion of the LA in the 2ch and 4ch views were extracted from the Circle CVI^42^ outputs. To validate the TSFFD methods for 3D feature tracking of the LA using cine MR stacks, we extracted the corresponding contours of the LA in the 2ch and 4ch views at their respective slice locations. The minimum distance for each point in the Circle CVI^42^ contour to the mesh derived contour was calculated. The RMSE of the contour difference was averaged across the LAX cine time points for each case. We validated the TSFFD method on CMR images comparing the 4ch and 2ch deformations predicted by the cine MR image registration against the long axis contours automatically segmented via Circle CVI^42^ for the healthy and DCM cohort. The 4ch and 2ch viewers were unavailable for the AF patient cohort. We found that the mean error at all time points was <3.0mm (healthy: 2.4±1.6mm; DCM: 2.7±0.7). The accuracy of the verification and validation were below the slice thickness of the cine MR images used in the image registration.


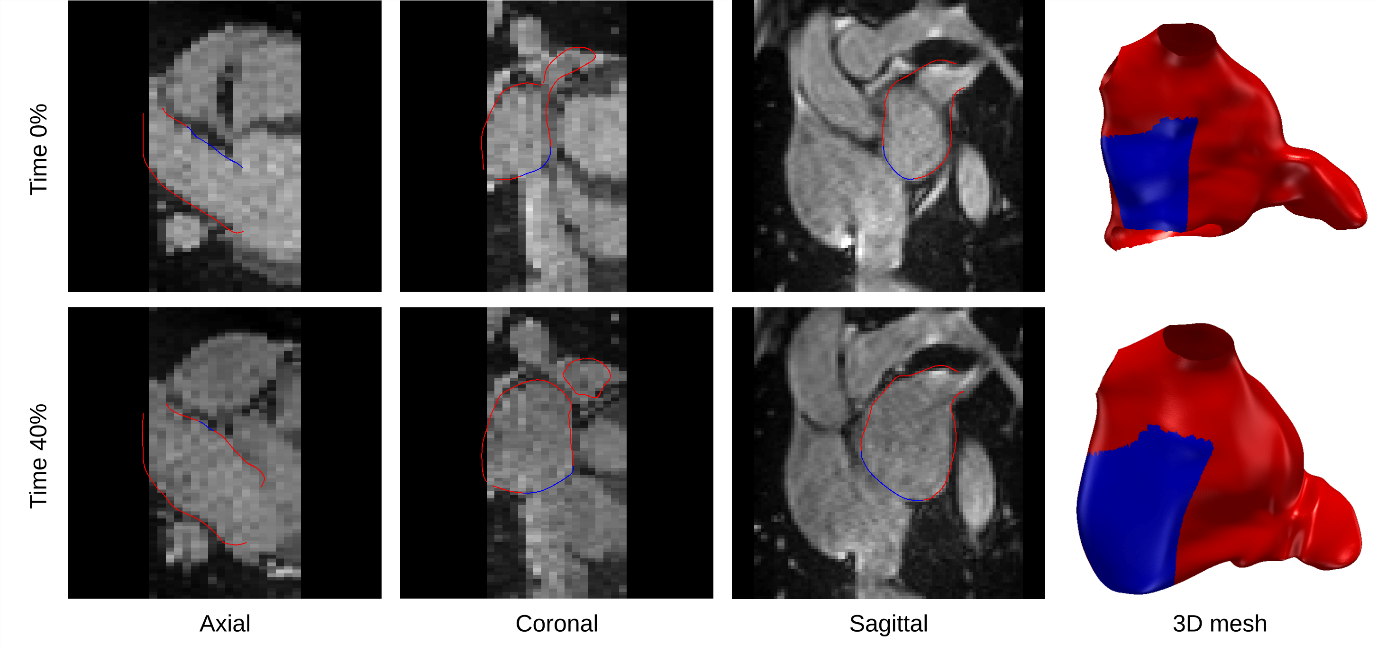


Figure 1: The feature tracking algorithm was used to track the deformations of the atria throughout the cardiac cycle. The LA mesh (with the septal region highlighted in blue) is overlaid on the short axis 3D cine MR images at LVED (0%) and LVES (40%) timepoints.

**4: Analysis of the regional strain: Healthy vs AF**

In this section, we present the results for the direct and indirect comparison of the area strains, as given by the change in area in each region in the LA with respect to LVED, in the healthy and AF cohorts. In addition to area strain, we assessed fiber-directional strain to capture the influence of myocardial fiber orientation on regional deformation. Fiber directions, which align with the principal axes of stretch and contraction, may provide complementary information on tissue mechanics. Endocardial and epicardial fiber fields were mapped from an averaged human atlas using UACs^6^, and fiber strains were computed accordingly. While area strain reflects changes in surface area (i.e., in-plane deformation), fiber strain quantifies deformation along a single fiber axis. The consistently higher area strain values likely reflect additional cross-fiber deformation not captured in the fiber-aligned strain measures.

Statistical analyses were performed using the python scipy (1.18.0) and statsmodel (0.14.0) libraries. One-way ANOVA showed that the area strain was significantly heterogeneous across the regions (p-value<0.001) in both the healthy and AF patient cohorts. Post-hoc Tukey tests showed that there was significantly higher area strain in the Septal region (p-value<0.05) in both cohorts (Figure 2). The largest and smallest relative strains were in the septal and the PVAs (RPVA for the healthy and LPVA for the AF), respectively (Table 1).

A mixed effects model was used to compare the area strain for the regions across the healthy and AF cohorts and our results showed that both region and cohort classification significantly affect area strain (Figure 3). A mixed effects model was used here as area strain was measured across different regions in each patient, so in this case the region would be the within-subject factor and the cohort would be the between-subject factor. Our results showed that the overall regional area strain was significantly higher in the healthy cohort (p-value<0.001) and that there was significant variation in the area strains across regions.

A)
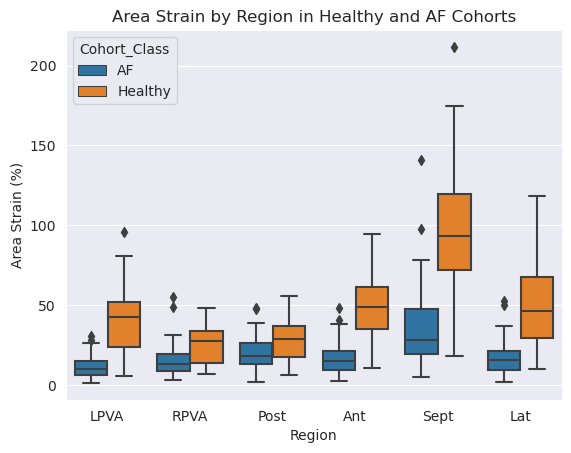


B)
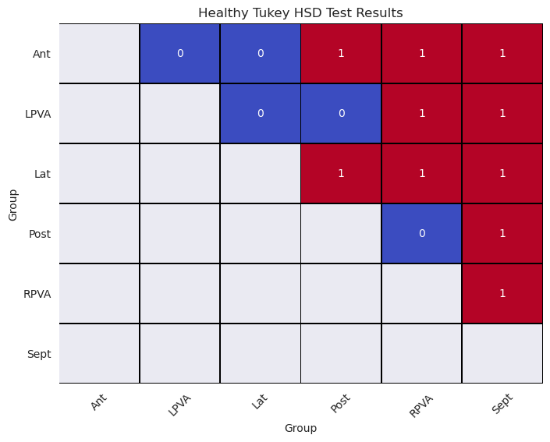

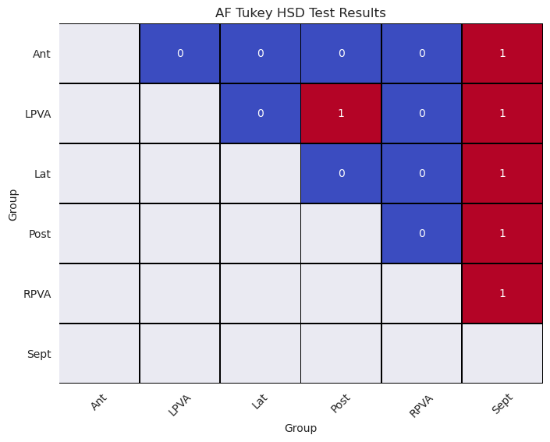


Figure 2: (A) Box plots for the comparison of the regional area reservoir strains between healthy volunteers and AF patients. (B) The regional area strains and the heat map comparison between the regions showing where a significant difference (1:red) or not (0:blue) exists between regions for the healthy and AF cohorts. LPVA: Left pulmonary veins antrum; RPVA: Right pulmonary veins antrum; Ant: Anterior wall; Post: Posterior wall; Sept: Septum; Lat: Left lateral wall.


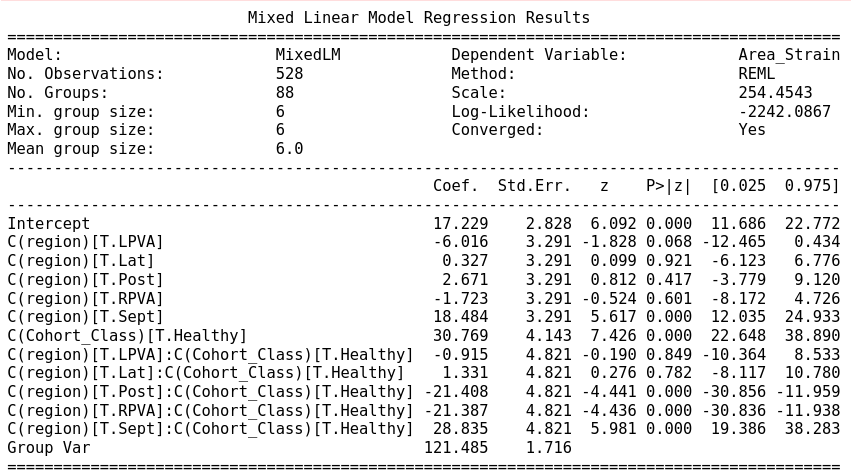


Figure 3: The mixed effects model results for assessing the impact of healthy vs AF and the regional location on the area strains.

Table 2: The regional area, endocardial fiber, and epicardial reservoir strain (RS) in each region for the AF patient, healthy volunteer, and DCM familial cohorts. LPVA: Left pulmonary veins antrum; RPVA: Right pulmonary veins antrum; Ant: Anterior wall; Post: Posterior wall; Sept: Septum; Lat: Left lateral wall

|  | Regions | | | | | |
| --- | --- | --- | --- | --- | --- | --- |
|  | **LVPA** | **RVPA** | **Post** | **Ant** | **Sept** | **Lat** |
| AF (n=47) |  |  |  |  |  |  |
| Area RS (%) | 11.2 ± 6.7% | 15.5 ± 10.4% | 19.9 ± 10.6% | 17.2 ± 10.6% | 35.7 ± 25.0% | 17.6 ± 11.3% |
| Endo fiber RS(%) | 8.0 ± 4.5 | 10.6 ± 7.2% | 11.4 ± 5.8% | 10.2 ± 5.9% | 20.3 ± 14.3% | 10.9 ± 8.2% |
| Epi fiber RS (%) | 8.0 ± 4.5 | 10.7 ± 7.3% | 11.5 ± 5.9% | 10.6 ± 6.5% | 20.3 ± 15.6% | 10.2 ± 7.5% |
| Healthy (n=41) |  |  |  |  |  |  |
| Area RS (%) | 41.1 ± 20.2% | 24.9 ± 12.0% | 29.3 ± 13.1% | 48.0 ± 19.8% | 95.3 ± 40.4% | 49.7 ± 26.3% |
| Endo fiber RS(%) | 24.7 ± 9.7% | 21.7 ± 9.3% | 19.5 ± 7.3% | 32.0 ± 11.8% | 57.8 ± 22.7% | 38.3 ± 27.4% |
| Epi fiber RS (%) | 25.6 ± 10.0% | 21.0 ± 9.0% | 17.7 ± 6.7% | 31.6 ± 12.4% | 56.7 ± 22.4% | 37.4 ± 24.9% |
| Familial DCM (n=31) |  |  |  |  |  |  |
| Area RS (%) | 35.8±14.8 | 27.9±18.6 | 33.0±18.2 | 45.6±22.0 | 75.3±35.2 | 48.6±31.6 |
| Endo fiber RS(%) | 24.6±8.8 | 27.3±13.4 | 26.5±11.3 | 36.7±17.2 | 47.5±22.5 | 40.5±23.0 |
| Epi fiber RS (%) | 26.5±9.8 | 25.8±12.5 | 24.3±10.3 | 35.4±16.0 | 51.5±23.1 | 38.9±22.5 |

**5: Regional comparisons of the AF fibrosis versus strain**

In this section we present the full analysis for the regional area strain measures versus the regional fibrosis using the AF cohort. We used the AF cohort data to assess the impact of the fibrosis burden and regional location on the area strain using a mixed effect model (Figure 4 A&B). We found that there was no significant difference (p-value>0.05) in the area strains with or without severe fibrosis, whether accounting for the regional locations (p-value>0.2) or not (p-value=0.774).

Principal component analysis (PCA) was used to decompose the strain curves into a lower dimensional space, using the sklearn python library,^7^ similar to the methods used by Schäfer et al.^8^ We found that the first 2 modes of the PCA explained >93% of the variance in the strain curves for the AF cohort. The 1st PC of the PCA was able to explain >69% of the variance and describes the strain magnitude, the 2nd PC accounts for >24% of the variance and describes the time to peak in the strain-time curves (Figure 4H). We repeated the same analysis as above and similarly found no significant effects of the fibrosis classification for the first and second principal components of the area strain (Figure 4 C&D, p-value>0.05) and for the strains in the epicardial and endocardial fiber directions (Figure 4 E&F, p-value>0.1).

The overall regional area strain was significantly lower in the AF patient cohort (p-value<0.001). To account for these physiological strain variations, we calculated the z-score of the regional strains in the AF patient cohort relative to the regional strains in the healthy volunteers. We repeated the statistical analysis with a mixed effects model to compare the regional area strains z-scores with the fibrosis classification in the AF cohort (Figure 4G). We found that the fibrosis burden had no significant effect on the relative area strain (p-value>0.6).

A)
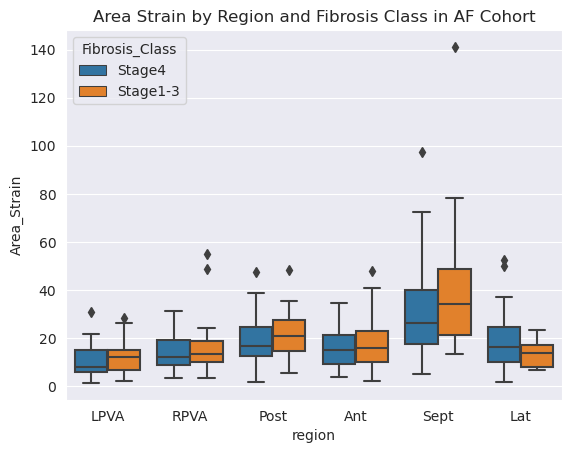


B)
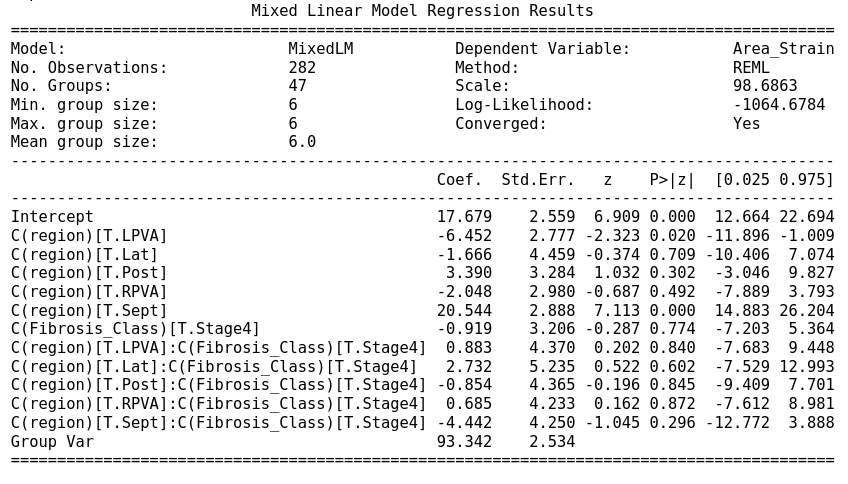


C)
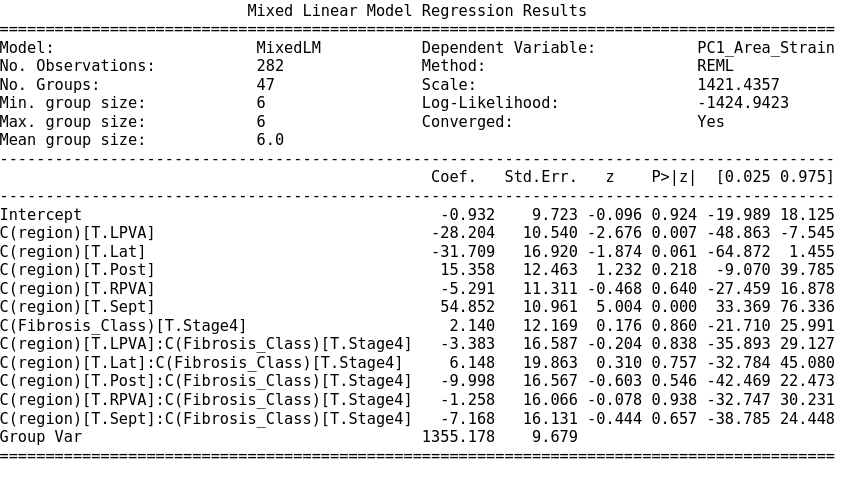


D)
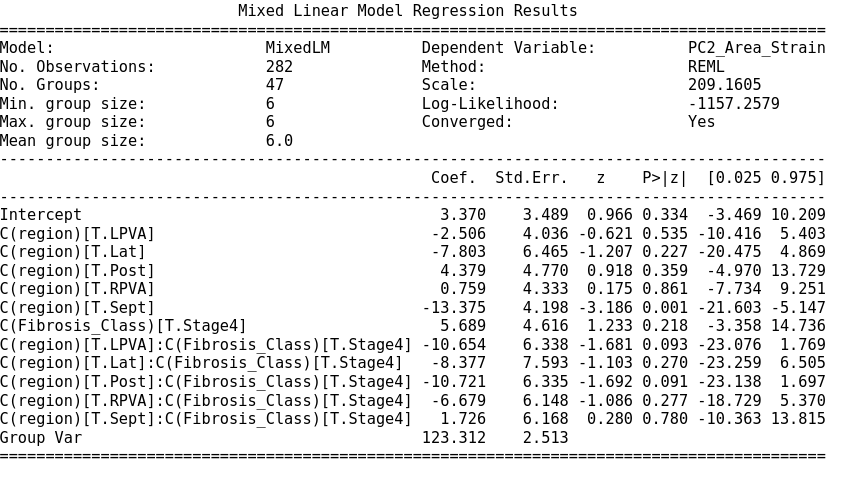


E)
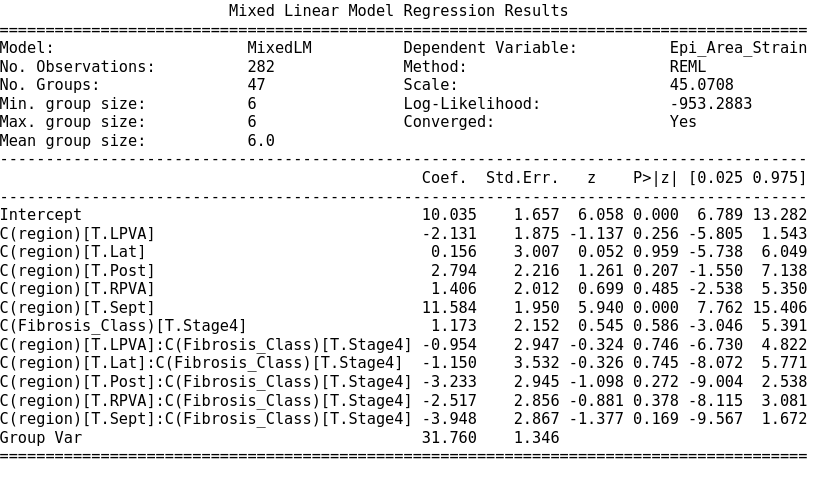


F)
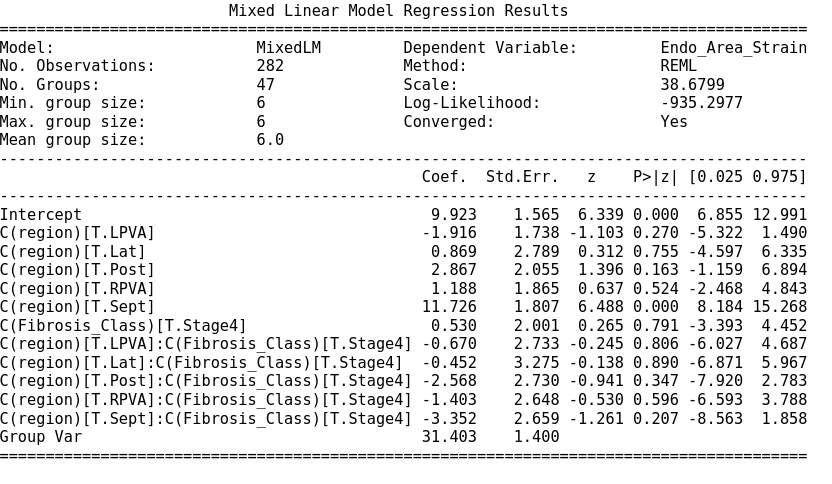


G)
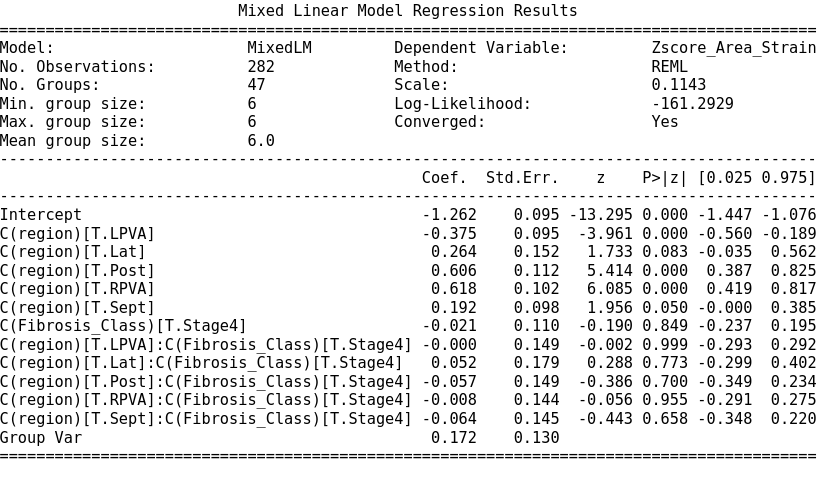


H)
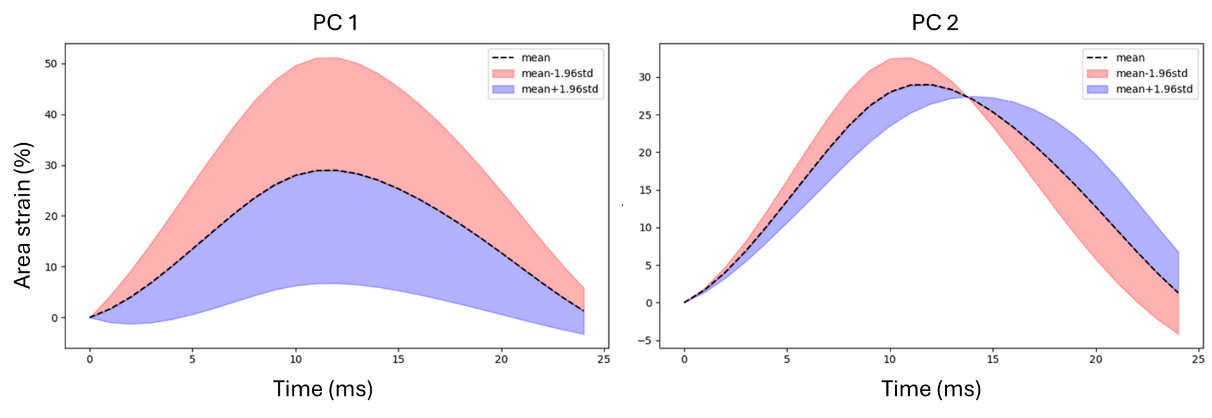


Figure 4: A) Area strain by fibrosis classification and region. The mixed effects model results for assessing the impact of the fibrosis classification (stage1-3 vs stage 4) and the regional location on the B) area strains, C) the first principal component (PC) of the area strain, D) the second PC of the area strain, E) strains along the epicardial fiber directions, and F) strains along the endocardial fiber directions, and G) normalised area strains. H) Mean and 95% confidence interval of PC1 and PC2 of the area strain. LPVA: Left pulmonary veins antrum; RPVA: Right pulmonary veins antrum; Ant: Anterior wall; Post: Posterior wall; Sept: Septum; Lat: Left lateral wall.

**6: Regional fibrosis analysis**

In this section, we present the full analysis for the regional distribution of fibrosis for two methods of subdividing the left atria. There is a consensus for the division of the left ventricle into subregions (16 or 17 segment AHA model), however there is currently no such agreement for the regional division of the left atria.

In the main manuscript, we divided the LA anatomy into 6 regions (Higuchi mapping), with the full analysis based on this regional mapping presented below (Table 3). Alternate divisions of the atria have been used by other authors, such as the Benito mapping with the atria divided into 12 region.^9^ In the Higuchi mapping system, the PV antra were defined as 10mm from the PV-LA body junction^10^, while the UACs were used to define the anterior and posterior walls from the roof of the atria to the LA floor bordered by the PV on the anterior and posterior faces of the LA. The septal wall was defined as the surface between the LA and RA, while the lateral wall is the free wall on the left side of the LA. Similarly, the UACs were used to define regions in the Benito mapping system as shown in Figure 6A. As a comparison of the effects of alternative mapping strategies, we have repeated the analysis in the Benito mapping system.

One-way ANOVA tests showed there was a heterogeneous regional fibrosis distribution in the left atria in both mapping strategies (p-value<0.001). Analyses with Tukey post-hoc tests are presented in Figure 4 and Table 3, and Figure 6 and Table 4 for the Higuchi and Benito mapping strategies, respectively.

In the Higuchi regional distribution, post-hoc analysis showed that the fibrosis burden was significantly (p-value<0.05) higher in the lateral region, in comparison to the LPVA, posterior and septal regions. The posterior region also had a significantly higher fibrosis burden in comparison to the LPVA region. The lowest and highest fibrosis burdens were in the LPVA and lateral regions, respectively.

In contrast, for the Benito mapping strategy, the post-hoc tests showed significantly (p-value<0.05) more fibrosis in the posterior wall 3, floor regions, anterior regions closer to the MV plane and the left lateral wall. The region with the lowest fibrosis burden was in the posterior wall 1, which like the Higuchi LPVA region, is situated close to the LSPV. The highest fibrosis burden was in the floor 5 region.

Broadly speaking, both mapping strategies indicated that there was significant fibrosis in the lateral and posterior regions (Higuchi posterior wall and Benito posterior wall 3) and with the lowest fibrosis around the LSPV. Different mapping strategies for the division of the atria means that there can be confusion as to the location of the atria that conclusions are being drawn about. For example, the posterior region in the Higuchi map is roughly equivalent to 4 regions in the Benito system (posterior walls 3 and 4, and floor 5 and 6), where floor 5 was the area with the highest fibrosis. A community standard for regions in the atria, like in the ventricle would facilitate comparisons across studies.

a)
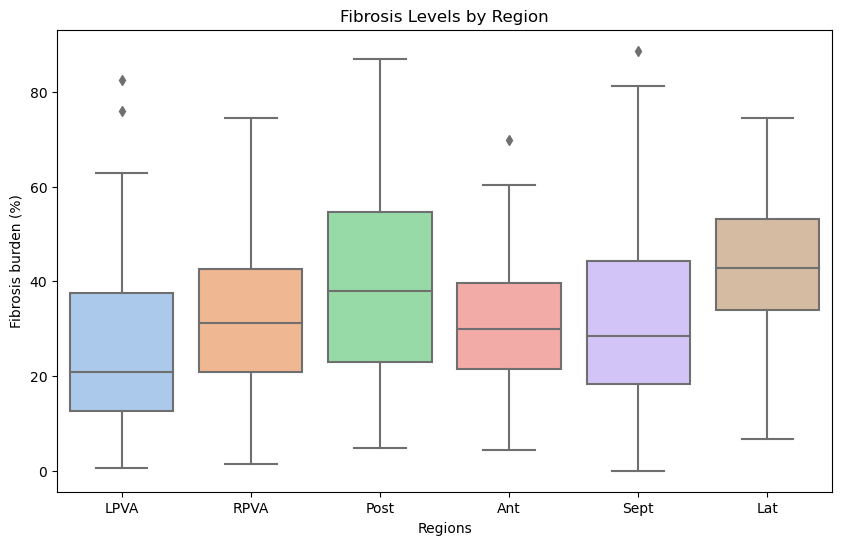
b)
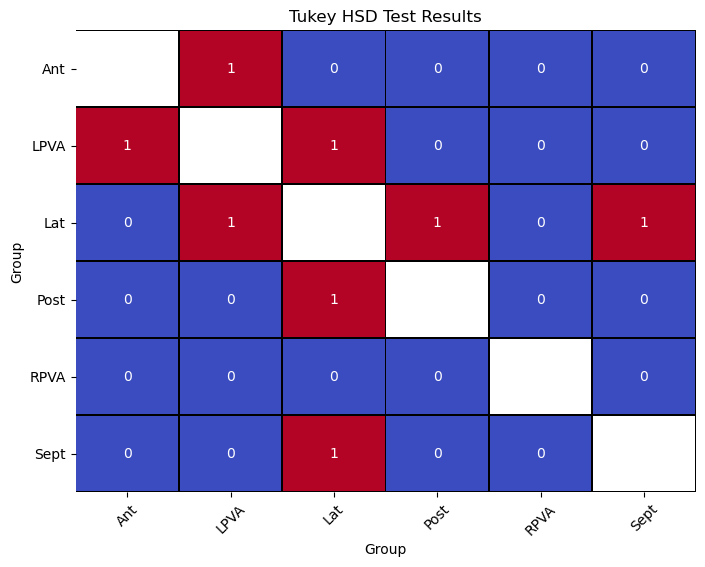


Figure 5: a) The fibrosis burden for each region for the AF cohort. b) Each pair of regions were compared to identify which regions had a significant difference between them (red 1: p-value≤0.05, blue 0: p-value>0.05). LPVA: Left pulmonary veins antrum; RPVA: Right pulmonary veins antrum; Ant: Anterior wall; Post: Posterior wall; Sept: Septum; Lat: Left lateral wall.

Table 3: Fibrosis burden in each of the Higuchi defined regions for the AF cohort

|  | Fibrosis burden (mean ± SD) (%) | Cases with severe fibrosis (%) |
| --- | --- | --- |
| LPVA | 26.0 ± 18.5 | 36.2 |
| RPVA | 33.4 ± 17.0 | 53.2 |
| Post | 40.9 ± 21.8 | 66.0 |
| Ant | 31.4 ± 15.0 | 48.9 |
| Sept | 32.8 ± 20.1 | 46.8 |
| Lat | 44.4 ± 17.2 | 85.1 |

A)
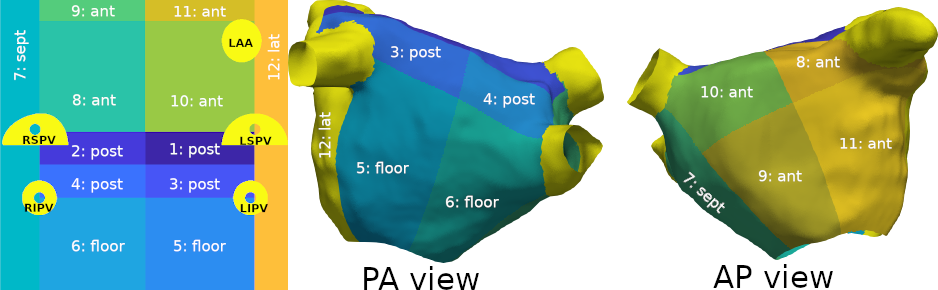


B)
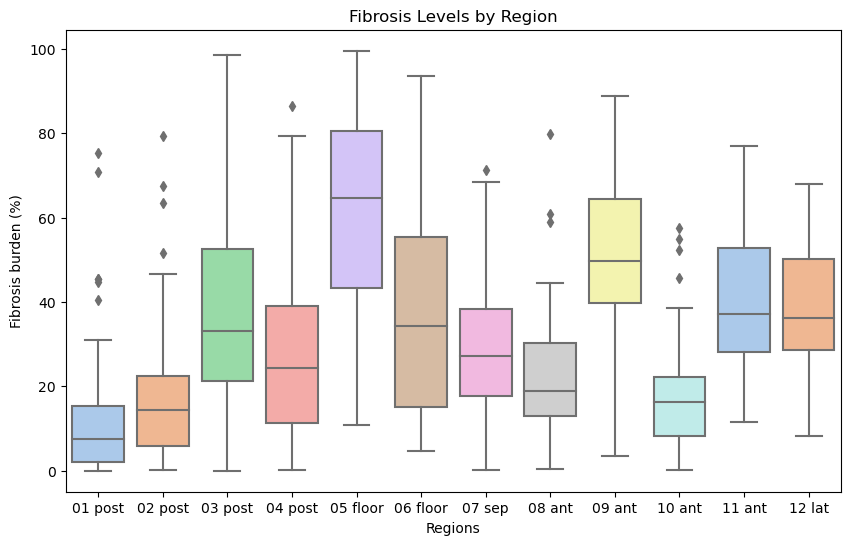


C)
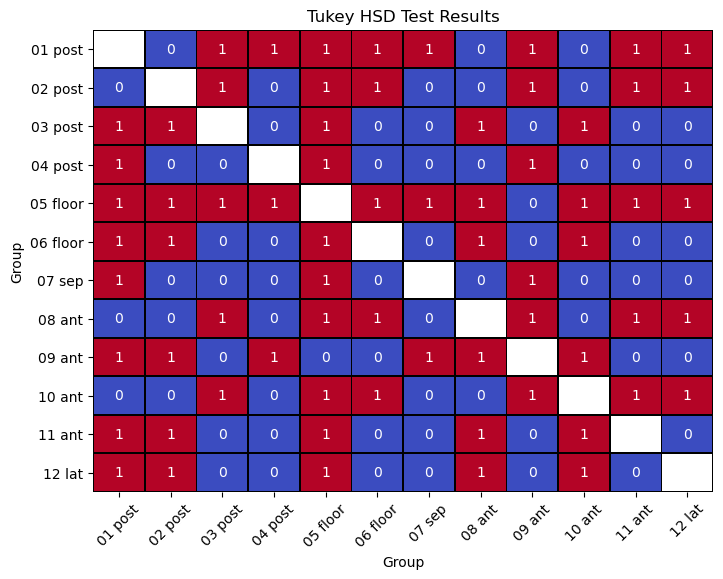


Figure 6: A) The Benito 12 region mapping of the atria in the main manuscript, with the universal atrial coordinates (UAC) map, posterior anterior (PA) and Anterior posterior views. B) We plotted the fibrosis burden for each region and C) compared each pair of regions to identify which regions had a significant difference between them (red 1: p-value≤0.05, blue 0: p-value>0.05). Post: Posterior wall; Sep: Septal wall; Ant: Anterior wall; Lat: Left lateral wall.

Table 4: The fibrosis burden for each of the Benito subregions in the AF cohort. Post: Posterior wall; Sep: Septal wall; Ant: Anterior wall; Lat: Left lateral wall.

| **Region** | **Fibrosis burden**  **(mean ± SD) (%)** | **Cases with severe fibrosis (%)** |
| --- | --- | --- |
| **1 Posterior wall** | 14.0 ± 17.7% | 14.9 |
| **2 Posterior wall** | 18.2 ± 18.3% | 17.0 |
| **3 Posterior wall** | 38.8 ± 23.7% | 61.7 |
| **4 Posterior wall** | 27.5 ± 21.5% | 38.3 |
| **5 Floor** | 60.2 ± 24.7% | 85.1 |
| **6 Floor** | 37.3 ± 25.8% | 57.4 |
| **7 Septal wall** | 29.2 ± 17.3% | 44.7 |
| **8 Anterior wall** | 22.8 ± 15.8% | 25.5 |
| **9 Anterior wall** | 49.2 ± 21.4% | 80.9 |
| **10 Anterior wall** | 18.4 ± 14.2% | 19.1 |
| **11 Anterior wall** | 40.7 ± 17.4% | 66.0 |
| **12 Left lateral wall** | 38.1 ± 13.9% | 70.2 |

**7: Machine learning models**

Machine learning models were used to assess the ability of the regional (region, area strain and strain rate, fiber strains) and global (area RS, LAV, and LAEF) features in classifying severe regional fibrosis in the AF cohort. We tested Random Forest, Logistic Regression and AdaBoost algorithms from the scikit-learn library (1.3.0) and found that the Random Forest technique gave the best results. In the tables below, we have presented the accuracy of the five-fold cross validation, ROC AUC and the p-values for the classifier and the ROC AUC curves for the 3 types of machine learning techniques used. A non-significant p-value (p-value>0.05) indicates that the classifier performs no better than random chance. In the tables below we have highlighted the classifiers that perform better than random chance [Tables 5-7].

All the logistic regression classifiers do not perform better than random chance. While the AdaBoost classifiers based on the global features (area RS, LAV, and LAEF) do perform better than random chance, the accuracy of these models were less than the corresponding Random Forest classifiers. In the Random Forest classifiers, we found that in addition to the global features, regional features (region, PC2 of the area and fiber strains) also performed better than random chance. PC2 (time to peak) reflects the timing of regional contraction, while PC1 (amplitude) indicates contraction magnitude. This may reflect the higher baseline variation in strain amplitude, as measured by PC1, compared to the lower variation in PC2, making changes in PC2 potentially more sensitive to local fibrosis burden. In the main manuscript, we have reported machine learning results based on the Random Forest classifiers.

*Table 5: Regional and global features were used to predict severe fibrosis for the atrial fibrillation cohort (p-values<0.05 in bold) with a* ***Logistic Regression*** *model.*

|  | **Five-fold cross**  **validation accuracy** | **Classifier**  **p-value** | **ROC AUC** | **ROC AUC**  **p-value** |
| --- | --- | --- | --- | --- |
| *Regional* |  |  |  |  |
| **Region** | 59.5 ± 7.1% | 0.055 | 0.558 | 0.228 |
| **Area RS** | 55.7 ± 2.3% | 0.008 | 0.563 | 0.202 |
| **Normalised Area RS** | 53.9 ± 2.2% | 0.023 | 0.539 | 0.305 |
| **Area strain rate** | 56.0 ± 0.7% | <0.001 | 0.349 | 0.969 |
| **Endo fiber RS** | 56.4 ± 2.5% | 0.011 | 0.571 | 0.169 |
| **Epi fiber RS** | 56.0 ± 2.1% | 0.003 | 0.575 | 0.176 |
| **Area PC1** | 55.7 ± 1.8% | 0.003 | 0.581 | 0.163 |
| **Area PC2** | 56.7 ± 0.5% | <0.001 | 0.458 | 0.726 |
| **Endo fiber PC1** | 56.4 ± 2.0% | 0.003 | 0.597 | 0.110 |
| **Endo fiber PC2** | 56.0 ± 0.7% | 0.003 | 0.440 | 0.791 |
| **Epi fiber PC1** | 54.2 ± 3.6% | 0.077 | 0.589 | 0.120 |
| **Epi fiber PC2** | 56.0 ± 0.7% | <0.001 | 0.539 | 0.319 |
| *Global* |  |  |  |  |
| **3D LAEF** | 56.4 ± 1.6% | 0.001 | 0.582 | 0.153 |
| **3D LAV** | 56.0 ± 0.7% | <0.001 | 0.371 | 0.953 |
| **Area RS** | 57.8 ± 1.1% | <0.001 | 0.662 | 0.019 |

*Table 6: Regional and global features were used to predict severe fibrosis for the atrial fibrillation cohort (p-values<0.05 in bold) with a* ***AdaBoost model.***

|  | **Five-fold cross**  **validation accuracy** | **Classifier**  **p-value** | **ROC AUC** | **ROC AUC p-value** |
| --- | --- | --- | --- | --- |
| *Regional* |  |  |  |  |
| **Region** | 57.8 ± 3.0% | 0.006 | 0.601 | 0.110 |
| **Area RS** | 54.6 ± 2.8% | 0.031 | 0.484 | 0.540 |
| **Normalised Area RS** | 56.0 ± 0.7% | <0.001 | 0.444 | 0.759 |
| **Area strain rate** | 55.3 ± 1.5% | 0.002 | 0.408 | 0.897 |
| **Endo fiber RS** | 53.2 ± 4.8% | 0.253 | 0.566 | 0.135 |
| **Epi fiber RS** | 55.0 ± 0.9% | <0.001 | 0.526 | 0.343 |
| **Area PC1** | 56.4 ± 1.5% | 0.001 | 0.515 | 0.407 |
| **Area PC2** | 56.4 ± 4.1% | 0.037 | 0.563 | 0.218 |
| **Endo fiber PC1** | 52.8 ± 5.1% | 0.331 | 0.579 | 0.102 |
| **Endo fiber PC2** | 53.9 ± 4.0% | 0.123 | 0.492 | 0.557 |
| **Epi fiber PC1** | 53.6 ± 5.4% | 0.258 | 0.388 | 0.923 |
| **Epi fiber PC2** | 54.6 ± 4.2% | 0.096 | 0.511 | 0.468 |
| *Global* |  |  |  |  |
| **3D LAEF** | **74.1 ± 5.0%** | **0.001** | **0.766** | **0.002** |
| **3D LAV** | **75.2 ± 6.2%** | **0.001** | **0.807** | **0.001** |
| **Area RS** | **75.5 ± 3.5%** | **<0.001** | **0.815** | **0.001** |

Table 7: Regional and global features were used to predict severe fibrosis for the atrial fibrillation cohort (p-values<0.05 in bold) with a **Random Forest** model.

|  | **Five-fold cross**  **validation accuracy** | **Classifier p-value** | **ROC AUC** | **ROC AUC p-value** |
| --- | --- | --- | --- | --- |
| *Regional* |  |  |  |  |
| **Region** | **56.0 ± 4.0%** | **0.044** | **0.638** | **0.027** |
| **Area RS** | 53.2 ± 5.4% | 0.307 | 0.506 | 0.500 |
| **Normalised Area RS** | 53.2 ± 6.0% | 0.345 | 0.491 | 0.553 |
| **Area strain rate** | 45.3 ± 6.7% | 0.792 | 0.441 | 0.763 |
| **Endo fiber RS** | 51.8 ± 7.5% | 0.661 | 0.504 | 0.490 |
| **Epi fiber RS** | 51.1 ± 3.9% | 0.621 | 0.59 | 0.119 |
| **Area PC1** | 49.0 ± 7.0% | 0.860 | 0.361 | 0.974 |
| **Area PC2** | **56.0 ± 3.9%** | **0.036** | **0.666** | **0.018** |
| **Endo fiber PC1** | 50.0 ± 10.1% | 0.860 | 0.361 | 0.967 |
| **Endo fiber PC2** | **51.0 ± 5.4%** | **0.036** | **0.666** | **0.019** |
| **Epi fiber PC1** | 56.0 ± 9.4% | 0.860 | 0.361 | 0.952 |
| **Epi fiber PC2** | **55.6 ± 9.2%** | **0.036** | **0.666** | **0.017** |
| *Global* |  |  |  |  |
| **3D LAEF** | **75.9 ± 5.8%** | **0.001** | **0.797** | **0.001** |
| **3D LAV** | **76.6 ± 7.2%** | **0.002** | **0.799** | **0.001** |
| **Area RS** | **74.8** **± 7.5%** | **0.003** | **0.811** | **0.001** |

**8. Fiber orientation**

The area reservoir strain is a measure of the non-directional LA enlargement. However, Suh et al 2008 showed that LA enlargement is not uniform, with dilation along the medial-lateral direction lower compared to longitudinal and anterior-posterior directions. ^11^ Longitudinal strain has also been shown to be a more informative measure than circumferential or radial strain in the LV.^12^ We posited that fiber strain could potentially be even more informative with larger strains occurring along the fiber axes as the atria enlarges.

Endocardial and epicardial fiber fields derived from a human atlas were mapped via UACs to test the effects of fiber directions on strain. ^6^ While a rule-based method^13^ would have smoother fiber distributions than anatomy atlas based methods, Roney et al found a greater correlation between the individual fiber fields and the averaged fiber field than the Labarthe rule-based fiber field.^6^

The epicardial and endocardial fiber orientations used in the models were estimated from an averaged fiber orientations from 7 human hearts (comprising of normal, AF and MI patients) scanned with via sub-millimeter diffusion tensor imaging. ^6,14^ Cine CMR were used to track the motion of the atria throughout the cardiac cycle using image with an interslice thickness of 4-8mm. We then averaged fiber strain measures over anatomical reigns (RPVA, LPVA, Septal, Inferior, Anterior, Posterior) to account for the resolution in the CMR images and potential noise in the fiber orientation estimates.

While disease can potentially modify fiber orientation in the left atria, Pashakhanloo et al found no obvious differences in the fiber architecture of the AF patients compared to the rest of the hearts. ^15^ While Roney et al found that fiber field 1 was the optimal map to use for arrhythmia simulations in the LA. We tested if variations in the fiber orientations or disease type could potentially alter the accuracy of fiber strain in classifying regions of severe fibrosis in AF patients with Random Forest models. The p-value of the ROC AUC curves were all >0.1, indicating that the classifiers based on regional fiber strain did not classify regions of severe fibrosis better than pure chance as shown in the table below.

Table 8: Fiber strain from anatomical atlas maps of the endocardial (endo) and epicardial (epi) fiber orientations were used to predict severe fibrosis in the atrial fibrillation cohort with a Random Forest model

| Fiber Field Map | Age/Sex | Cardiac Disease Status | 5 fold cross validation accuracy | classifier p-value | ROC AUC | ROC AUC p-value |
| --- | --- | --- | --- | --- | --- | --- |
| Endo average |  |  | 51.8 ± 7.5 | 0.32 | 0.504 | 0.491 |
| Endo 1 | 93/F | MI | 44.7 ± 4.4 | 0.075 | 0.494 | 0.53 |
| Endo 2 | 67/F | MI | 52.8 ± 3.7 | 0.205 | 0.597 | 0.123 |
| Endo 3 | 90/F | Normal | 49.7 ± 5.8 | 0.917 | 0.396 | 0.895 |
| Endo 4 | 76/F | Normal | 57.1 ± 4.6 | 0.036 | 0.479 | 0.593 |
| Endo 5 | 94/F | AF | 53.6 ± 3.8 | 0.137 | 0.484 | 0.584 |
| Endo 6 | 86/M | AF | 48.9 ± 2.6 | 0.455 | 0.541 | 0.298 |
| Endo 7 | 55/M | Normal | 61.0 ± 6.1 | 0.022 | 0.537 | 0.336 |
|  |  |  |  |  |  |  |
| Epi average |  |  | 51.1 ± 3.9 | 0.07 | 0.59 | 0.119 |
| Epi 1 | 93/F | MI | 49.3 ± 1.9 | 0.495 | 0.419 | 0.842 |
| Epi 2 | 67/F | MI | 51.1 ± 2.2 | 0.388 | 0.501 | 0.494 |
| Epi 3 | 90/F | Normal | 50.3 ± 4.2 | 0.881 | 0.444 | 0.745 |
| Epi 4 | 76/F | Normal | 56.0 ± 6.1 | 0.117 | 0.471 | 0.609 |
| Epi 5 | 94/F | AF | 49.3 ± 4.7 | 0.782 | 0.468 | 0.643 |
| Epi 6 | 86/M | AF | 54.3 ± 5.2 | 0.178 | 0.476 | 0.613 |
| Epi 7 | 55/M | Normal | 51.4 ± 7.4 | 0.724 | 0.404 | 0.881 |

**References**

1. January CT, Wann LS, Alpert JS, et al. 2014 AHA/ACC/HRS Guideline for the Management of Patients With Atrial Fibrillation: Executive Summary. *J Am Coll Cardiol*. 2014;64(21). doi:10.1016/j.jacc.2014.03.021

2. den Uijl DW, Cabanelas N, Benito EM, et al. Impact of left atrial volume, sphericity, and fibrosis on the outcome of catheter ablation for atrial fibrillation. *J Cardiovasc Electrophysiol*. 2018;29(5):740-746. doi:10.1111/jce.13482

3. van de Vegte YJ, Siland JE, Rienstra M, van der Harst P. Atrial fibrillation and left atrial size and function: a Mendelian randomization study. *Sci Rep*. 2021;11(1). doi:10.1038/s41598-021-87859-8

4. Seewöster T, Spampinato RA, Sommer P, et al. Left atrial size and total atrial emptying fraction in atrial fibrillation progression. *Heart Rhythm*. 2019;16(11):1605-1610. doi:10.1016/j.hrthm.2019.06.014

5. Sillett C, Razeghi O, Lee AWC, et al. A three-dimensional left atrial motion estimation from retrospective gated computed tomography: application in heart failure patients with atrial fibrillation. *Front Cardiovasc Med*. 2024;11. doi:10.3389/fcvm.2024.1359715

6. Roney CH, Bendikas R, Pashakhanloo F, et al. Constructing a Human Atrial Fibre Atlas. *Ann Biomed Eng*. 2021;49(1). doi:10.1007/s10439-020-02525-w

7. Pedregosa F, Varoquaux G, Gramfort A, et al. Scikit-learn: Machine learning in Python. *the Journal of machine Learning research*. 2011;12:2825-2830.

8. Schäfer M, Mitchell MB, Frank BS, et al. Myocardial strain-curve deformation patterns after Fontan operation. *Sci Rep*. 2023;13(1):11912. doi:10.1038/s41598-023-39226-y

9. Benito EM, Cabanelas N, Nuñez-Garcia M, et al. Preferential regional distribution of atrial fibrosis in posterior wall around left inferior pulmonary vein as identified by late gadolinium enhancement cardiac magnetic resonance in patients with atrial fibrillation. *Europace*. 2018;20(12):1959-1965. doi:10.1093/europace/euy095

10. Higuchi K, Cates J, Gardner G, et al. The Spatial Distribution of Late Gadolinium Enhancement of Left Atrial Magnetic Resonance Imaging in Patients With Atrial Fibrillation. *JACC Clin Electrophysiol*. 2018;4(1):49-58. doi:10.1016/j.jacep.2017.07.016

11. Suh IW, Song JM, Lee EY, et al. Left Atrial Volume Measured by Real-Time 3-Dimensional Echocardiography Predicts Clinical Outcomes in Patients with Severe Left Ventricular Dysfunction and in Sinus Rhythm. *Journal of the American Society of Echocardiography*. 2008;21(5). doi:10.1016/j.echo.2007.09.002

12. Fung MJ, Leung DY, Thomas L. Differential Myocardial Fibre Involvement by Strain Analysis in Patients With Aortic Stenosis. *Heart Lung Circ*. 2018;27(11):1357-1367. doi:10.1016/J.HLC.2017.08.017

13. Labarthe S, Bayer J, Coudière Y, et al. A bilayer model of human atria:mathematical background, construction, and assessment. *Europace*. 2014;16. doi:10.1093/europace/euu256

14. Pashakhanloo F, Herzka DA, Ashikaga H, et al. Myofiber architecture of the human atria as revealed by submillimeter diffusion tensor imaging. *Circ Arrhythm Electrophysiol*. 2016;9(4). doi:10.1161/CIRCEP.116.004133
